# Strengthening Anti-Racist Gerontology Research: Does One Size Fit All?

**DOI:** 10.1093/geroni/igab046.910

**Published:** 2021-12-17

**Authors:** Lyn Holley

**Affiliations:** University of Nebraska at Omaha, Omaha, Nebraska, United States

## Abstract

This paper is based on a scoping review and conceptual analysis of research literature about incorporating anti-racism into social science research practices. In his examination of how anti-racist research can effectively borrow key concepts such as “validation and “reliability” from traditional social science research, Dei concludes that these concepts must be reconsidered to addresses the main issues of anti-racism. (2005). A further critique of these concepts is that they do not account for differences among racism as it is applied to different minoritized groups. Public Health Nurses and other practitioners have long recognized the importance of understanding and taking these differences into account in their “culturally competent” practice. (Lipscomb, Culture Care) Although there is some literature about de-centering whiteness in research (e.g., https://libguides.umn.edu/antiracismlens ), little is available to guide research that acknowledges and addresses overlapping yet differing contours of racism as experienced by different “races”, e.g., Black-Americans, Native American Indians”.

